# Dual-channel feature fusion network for sheep diseases question classification

**DOI:** 10.1371/journal.pone.0343990

**Published:** 2026-03-30

**Authors:** Gulizada Haisa, Gulimila Kezierbieke

**Affiliations:** College of Computer and Information Engineering, Xinjiang Agricultural University, Urumqi, China; Eskisehir Osmangazi University: Eskisehir Osmangazi Universitesi, TÜRKIYE

## Abstract

To address the challenges of feature sparsity, semantic ambiguity, and insufficient feature extraction in sheep disease question classification, this paper proposes a novel model named Dual-Channel Feature Fusion Network for Sheep Diseases Question Classification (DFF-SDQC). The model leverages the CINO pre-trained model to generate dynamic word embeddings, thereby enriching semantic representations. Subsequently, global textual features are captured through BiLSTM, while deeper local contextual features are extracted using an attention mechanism. To further enhance the robustness and generalization of the model, a question-word attention mechanism is introduced, enabling the attention matrix to better capture the intentions expressed by interrogative words, thus strengthening the overall feature representation of the question. Finally, dual-channel feature information is fused to obtain the final textual representation. Experimental results on the D-SDQC and D-TQC datasets show that DFF-SDQC achieves an F1-score of 93.18% on D-SDQC, improving 2.22 percentage points over the strongest baseline, demonstrating the effectiveness of the dual-channel fusion and attention design.

## 1. Introduction

Question classification plays a pivotal role in question answering (QA) systems, as it enables mapping user queries to predefined categories for efficient answer retrieval [1]. Existing research on question classification has primarily focused on real-time queries such as temporal, entity-based, and descriptive questions. For example, the TREC conference [2] has long emphasized fact-based question classification. In contrast, domain-specific QA tasks involve not only factual queries but also large amounts of professional, knowledge-intensive questions. In high-resource languages such as English [3] and Chinese [4], numerous studies have been proposed to improve the effectiveness of question classification models. While existing research has achieved considerable progress in high-resource languages such as English and Chinese, low-resource languages like Kazakh remain underexplored, facing challenges such as data sparsity, morphological complexity, and insufficient corpus resources.

To address these challenges, we propose the Dual-Channel Feature Fusion Network for Sheep Diseases Question Classification (DFF-SDQC), tailored for the veterinary domain. Unlike traditional approaches that struggle with sparse and ambiguous short-text representations, DFF-SDQC integrates dynamic word embeddings from a pre-trained CINO model with bidirectional sequence features from BiLSTM. In addition, a novel question-word attention mechanism is designed to capture the intent expressed by interrogatives, while an enhanced convolutional layer strengthens global and contextual feature representations. Furthermore, we construct a domain-specific dataset for sheep disease question classification in Kazakh, covering 32 distinct question types. Experimental results show that DFF-SDQC consistently outperforms competitive baselines, highlighting its robustness and generalization ability in both low-resource and specialized domains.

The remainder of this paper is structured as follows. The subsequent section provides an overview of related work. Section 3 presents our proposed model. Section 4 outlines our experimental setup, including datasets, baselines, implementation details, experimental results, and analysis. Finally, Section 5 concludes the paper and discusses future directions.

## 2. Related work

There are a lot of question classification tasks and approaches, and we brieﬂy review the most widely-used methods in this paper.

Question classification is a fundamental text classification task with significant applications in natural language processing (NLP) fields such as question answering (QA) and dialogue systems. The earliest approaches were rule-based, where a predefined set of rules guided the extraction of semantic information from text. While these methods could achieve satisfactory classification results, they required extensive handcrafted rules and exhibited poor generalization. For example, Hovy et al.[5] employed rule-based strategies to represent text with handcrafted rules for classification, and Brill et al. [6] applied regular expressions for text classification. However, such approaches were inherently limited by subjective human judgments.

Traditional machine learning methods alleviated the dependence on handcrafted rules by leveraging larger corpora or iterative optimization, though they still required large-scale annotated datasets. Metzler et al.[7] applied radial basis kernel functions combined with multiple feature fusion techniques for English question classification, while Zhang et al.[8] introduced tree kernels to allow support vector machines to exploit syntactic structures of questions. Nevertheless, these methods still relied heavily on manual feature engineering, which constrained their scalability and efficiency.

With the rapid advancement of NLP, many scholars have turned to deep learning techniques to enhance the performance of question classification [9]. Kim et al.[10] proposed a convolutional neural network (CNN)-based sentence classification model using word embeddings. Dachapally et al.[11] extended CNN architectures by first classifying questions into broad categories and then refining them into more specific classes using prior knowledge. In the Chinese question classification domain, Liu et al. [4] employed attention mechanisms over BiGRU outputs to assign weights and used CNNs to learn local feature representations, effectively combining the strengths of BiGRU and CNN. Similarly, Shi et al. [12]adopted BiLSTM and CNN models for community QA question classification tasks.

More recently, studies have explored fine-tuning pre-trained models [13,14], multi-task learning [15,16], and knowledge distillation [17] to further improve classification accuracy. In addition, research in specialized domains has increasingly incorporated multimodal information [18,19] to overcome the limitations of unimodal approaches, thereby offering new directions and solutions for practical applications. For low-resource languages such as Kazakh, research on question classification remains limited. Wang et al. [20] pioneered the use of SVMs for Kazakh text classification, while Shaldan et al. [21] employed stem-based text representation combined with CNNs to classify Kazakh short texts. Haisa et al. [22,23] applied multi-feature deep learning approaches for Kazakh question classification, incorporating syntactic features. Despite these advances, substantial challenges remain in effectively modeling Kazakh question semantics due to resource scarcity and linguistic complexity. Overall, while numerous optimization algorithms and classification models have been proposed across different scenarios, question classification in domain-specific and low-resource settings remains an open challenge. In particular, for languages with limited data resources, such as Kazakh, question classification continues to be a difficult yet important research problem that requires further exploration.

## 3. Methodology

### 3.1. Problem definition

Natural language processing (NLP) for Kazakh remains a challenging task due to its agglutinative characteristics and rich morphological structures. In this study, we aim to effectively address the problem of Kazakh question classification by combining modern deep learning techniques with the linguistic characteristics of Kazakh interrogative structures.

On the one hand, Kazakh is recognized as a low-resource language [24,25], with highly complex linguistic features spanning five grammatical levels: syllables, stems, words, phrases, and sentences. Kazakh questions typically contain relatively few words, resulting in insufficient feature information for classification. Moreover, the addition of prefixes and suffixes to word stems produces numerous derived word forms. Stems serve as the core lexical units with semantic meaning, whereas affixes provide both semantic and grammatical functions [26]. Through morphological analysis and processing, meaningful and effective textual features can be preserved while reducing complexity and dimensionality. For example, the words “قويلار” (many sheep), “قويدىڭ” (of the sheep), “قويشى” (shepherd), “قويعا” (to the sheep), and “قويىڭ” (your sheep) all share the stem “قوي” (sheep), while the affixes “لار”, “دىڭ”, “شى”, “عا”, and “ىڭ” modify the semantics or grammatical roles. On the other hand, interrogative pronouns in Kazakh play a crucial role in indicating the type of information being sought. For instance, the interrogative pronouns “قانداي” (what kind) and “قانشالىقتا” (how long/how much) directly determine the type of the question.

In Table 1, the type of the question can be inferred primarily from the interrogative pronoun, indicating that the relationship between interrogative pronouns and other words has a decisive impact on question classification. To address these issues, this study first designs a classification schema for sheep disease-related questions and constructs a dataset through data augmentation. Then, for the Kazakh question classification task, we employ a pre-trained model to obtain dynamic word embeddings and introduce an interrogative pronoun attention mechanism to strengthen semantic feature representations of questions.

**Table 1:** Examples of Kazakh sheep disease questions and corresponding types.

Question (Kazakh)	English Translation	Interrogative Pronoun	Question Type
قويدىڭ سىرەسپەسى قانداي؟	What are the symptoms of sheep tetanus?	قانداي (what kind)	Symptom-related
قويدىڭ سىرەسىپەسى قانشالىقتا جازىلادى؟	How long does it take to cure sheep tetanus?	قانشالىقتا (how long)	Time-related

### 3.2. Dataset construction and augmentation

#### 3.2.1. Sheep disease question classification schema.

To construct a well-defined classification schema for sheep disease-related questions, it is essential to analyze existing research on sheep diseases [27–30] and conduct an in-depth investigation of sheep disease question corpora. Existing question classification frameworks generally fall into two categories: hierarchical (tree-structured) and flat (parallel) structures. Commonly used schemas often follow the UIUC classification system proposed for the TREC evaluation tasks [31]. However, studies focusing on veterinary medicine and sheep disease question classification are very limited. Therefore, in this work, we analyze the themes of user-generated questions from online sources and combine insights from relevant sheep disease literature to define a Kazakh-language question classification schema tailored to sheep diseases. The resulting dataset comprises **7 main categories and 32 subcategories**, with detailed information presented in Table 2.

**Table 2:** Examples of Kazakh sheep disease questions and corresponding types.

No.	Main Category	Subcategory
1	Disease Information	Disease Definition
2		Disease Causes
3		Disease Classification
4		Disease Stage
5	Symptoms	Disease Symptoms
6		Symptom Trends
7		Symptom-Related Factors
8	Diagnosis	Clinical Diagnosis
9		Required Diagnostics
10		Diagnostic Basis
11		Complications
12	Treatment	Treatment Methods
13		Recommended Drugs
14		Drug Dosage
15		Medication Timing
16		Treatment Efficacy
17	Prevention	Preventive Measures
18		Vaccine Types
19		Vaccination Timing
20		Vaccination Methods
21		Vaccine Efficacy
22		Vaccine Precautions
23	Husbandry Management	Environmental Hygiene
24		Feed and Water
25		Isolation and Quarantine
26		Seasonal Prevention
27		Preventive Guidelines
28		Breeding Experience
29	Emergency	First Aid Measures
30		Emergency Drugs
31		Isolation Measures
32		Transmission Routes

The Kazakh-language question classification schema for the sheep disease domain adopts a two-level hierarchical framework. The main categories primarily follow the UIUC English question classification standards, aligning with the principle that future research should build upon existing studies whenever possible. The subcategories are derived by analyzing users’ question patterns on various websites, enabling finer-grained categorization to facilitate subsequent answer extraction.

#### 3.2.2. Dataset augmentation.

Data augmentation is a widely used technique for expanding the size of training datasets, thereby enhancing the performance of deep learning models and mitigating overfitting. By increasing the diversity of training examples, data augmentation facilitates the development of more robust models, especially when training data is limited. In artificial intelligence, data augmentation methods have been extensively applied across various domains, including computer vision [32], speech processing [33], and natural language processing (NLP) tasks [34].

**Algorithm 1:** **Synonym Replacement for Data Augmentation**

1: Input: Question sets (seeds) Ds

2: Output: Augmented data Da

3: Initialize: Ds, Da

4: for m in range do

5: for k in range do

6:   Select k keywords from Ds

7:   Replace each keyword k with m synonyms

8:   Generate m new questions

9:  end for

10: end for

11: return Da

The synonym replacement method can generate additional training data, thereby improving model performance and robustness. The procedure is as follows:

1. **Preprocessing:** Clean the original dataset, compute the term frequency (TF) for each word in each category, and rank the words in descending order of TF to select k keywords.
2. **Synonym Dictionary Construction:** Create a dictionary containing m synonyms for each of the k keywords.
3. **Seed Selection:** Randomly select 30% of the questions in each category from the original dataset as seed data.
4. **Sentence Generation:** For each question in the seed set, extract all keywords and replace them with their synonyms from the dictionary to generate new sentences.

Following this process, the question dataset can be effectively augmented, improving model accuracy and generalization. Newly generated questions are labeled as valid if they are semantically and grammatically correct; otherwise, they are discarded. The selection of k keywords and m synonyms is illustrated in Algorithm 1.

Due to the scarcity of Kazakh synonym resources, we manually constructed a domain-specific synonym dictionary for the sheep disease domain. Two reference dictionaries were used during construction: (1) the Kazakh Synonym Dictionary [35], containing 16,000 entries, and (2) the Chinese-Kazakh Synonym Explanation Dictionary [36], containing 3,300 entries. After processing, a final dictionary comprising 1,000 words was created, with each word associated with 1–10 synonyms. This dictionary provides a valuable resource for Kazakh NLP tasks, particularly in domain-specific applications.

**Human verification and error rate control in synonym-based data augmentation:** To address the potential semantic drift and grammatical errors introduced by synonym replacement in low-resource language scenarios, this study incorporates a human verification mechanism into the data augmentation process to ensure the quality and reliability of the augmented data. In addition, the error rate induced by synonym replacement is systematically analyzed and quantitatively evaluated. After completing the synonym replacement procedure based on Algorithm 1, all automatically generated questions are first subjected to a manual verification stage. The verification is conducted by researchers proficient in the Kazakh language and familiar with the target domain, following a dual-criteria evaluation framework that simultaneously assesses semantic consistency and grammatical correctness. Specifically, (1) semantic consistency requires that the core meaning of an augmented question remains unchanged after keyword substitution and that the question still corresponds to its original category label; and (2) grammatical correctness requires that the generated question conforms to the fundamental morphological, word order, and syntactic rules of the Kazakh language. Only questions that satisfy both criteria are labeled as valid positive samples; otherwise, they are regarded as invalid and discarded.

To quantitatively evaluate the errors introduced by synonym replacement, we define a data augmentation accuracy metric t, which measures the proportion of valid samples among the augmented data, ast=P/A , where P denotes the number of augmented questions that pass manual verification in terms of both semantic and grammatical correctness, and A denotes the total number of questions generated during the data augmentation stage. Comparative analyses under different parameter configurations—namely, the number of selected keywords k and the number of synonyms m—indicate that, when k is fixed, increasing m leads to a gradual decrease in the proportion of valid samples. Conversely, when m is fixed, increasing k also results in a reduction in the proportion of valid samples. Relatively higher-quality augmented datasets are obtained when both k and m take smaller values.

Considering the trade-off between dataset expansion and the cost of manual verification, we ultimately select k=2 and m=2 as the optimal parameter configuration. Under this setting, the data augmentation accuracy remains at a high level while effectively increasing the number of training samples, thereby achieving a balance between data quality and data scale. These results demonstrate that the introduction of a human verification mechanism, together with a quantitative analysis of synonym replacement error rates, is a crucial factor in ensuring the feasibility and effectiveness of synonym-based data augmentation methods for low-resource languages.

#### 3.2.3. Sheep disease question classification dataset.

The sheep disease question classification dataset constructed in this study comprises two main components: the creation of the original dataset and its subsequent augmentation.

(1) Original Dataset Construction

First, raw question data were collected and organized from major websites and relevant literature, and categorized according to the proposed question classification schema. Subsequently, the collected questions were manually annotated by native Kazakh-speaking experts to ensure labeling accuracy and reliability. The original dataset consists of 3,000 questions distributed across 7 main categories and 32 subcategories, including: Disease Information (309 questions), Symptoms (314 questions), Diagnosis (421 questions), Treatment (571 questions), Prevention (593 questions), Husbandry Management (470 questions), and Emergency Care (322 questions). All annotation work was conducted manually to ensure high-quality labels.

(2) After Dataset Augmentation

**Fig 1:**
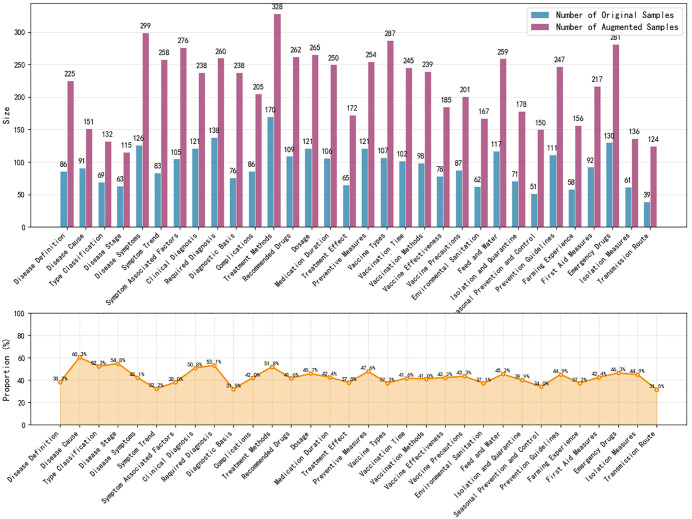
Distribution of sheep disease question classification dataset.

Using the data augmentation techniques described in Section 3.2.2, the original dataset was expanded to 7,000 questions. The distribution of question categories in the augmented dataset is shown in **Fig 1**. After augmentation, the distribution is as follows: Disease Information (623, 8.90%), Symptoms (843, 12.04%), Diagnosis (931, 13.30%), Treatment (1,275, 18.21%), Prevention (1,411, 20.16%), Husbandry Management (1,157, 16.53%), and Emergency Care (758, 10.83%). Data augmentation effectively increased the scale of training data, improving model generalization and the accuracy of question classification. The most frequent question categories in the dataset are Diagnosis, Treatment, Prevention, Symptoms, and Husbandry Management, whereas Disease Information and Emergency Care categories have the fewest questions. This distribution may reflect the data collection methods and the typical purposes for which users submit questions.

(3) Question Length Distribution

In natural language processing, text length is an important consideration because it affects both the complexity of semantic expression and the difficulty of comprehension. Specifically, in question classification tasks, longer questions generally contain more information, better reflecting the user’s intended meaning. Theoretically, as the number of words in a question increases, the provided contextual information becomes richer, enabling classification models to more accurately capture semantic features and assign correct categories.

**Fig 2:**
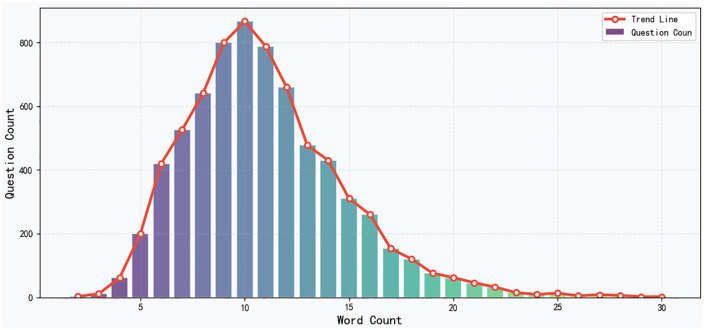
Distribution of question lengths in the question classification dataset.

Statistical analysis of the Kazakh-language question dataset shows that most questions contain between 2 and 29 words, with questions of 7–11 words being particularly prevalent. To more intuitively illustrate this trend, the word-count distribution is presented in **Fig 2**. The Fig clearly depict the relationship between the number of words in a question and the frequency of questions, providing valuable insight for model design and evaluation.

### 3.3. DFF-SDQC

**Fig 3:**
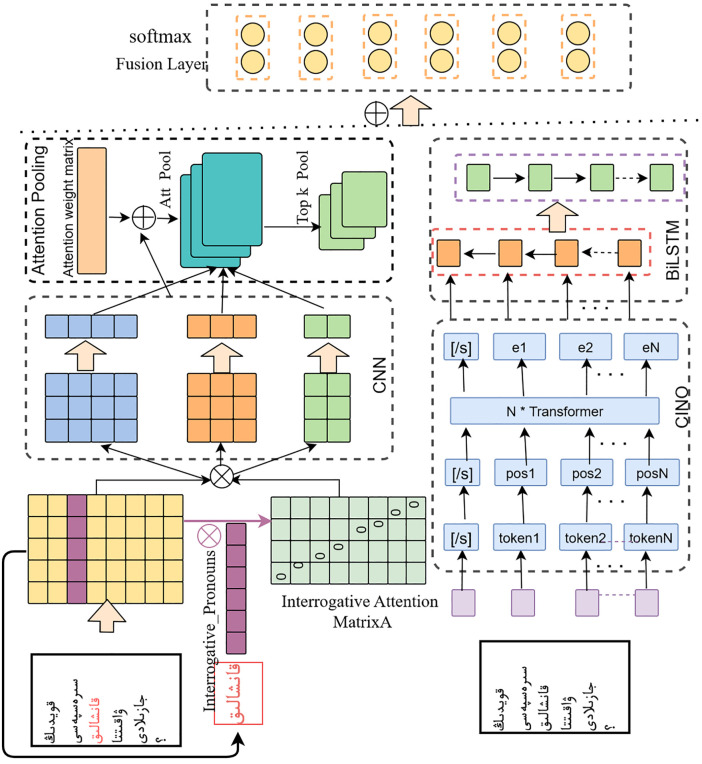
The model architecture.

The proposed Dual-Channel Feature Fusion Network for Sheep Diseases Question Classification (DFF-SDQC) is illustrated in Fig 3. Initially, the input question text undergoes tokenization and word embedding mapping. The processed text is then fed into the CINO model, which employs a multi-layer Transformer architecture to encode the text and extract semantic information. Leveraging large-scale pretraining, CINO effectively captures Kazakh semantic representations, producing word embeddings that encode lexical meaning, contextual information, syntactic and grammatical structures, as well as domain-specific knowledge. These embeddings are subsequently input into a bidirectional BiLSTM module, which combines forward and backward LSTM layers to capture bidirectional contextual information. Each LSTM direction processes the input sequence independently, thereby learning the temporal and sequential features of sheep disease questions. In the left channel, interrogative pronouns within the question are identified to construct an interrogative pronoun attention matrix. The resulting features are then fed into the left CNN channel, where convolutional kernels of various sizes extract multi-scale features. The attention mechanism emphasizes interrogative pronoun features, enabling the model to capture long-range dependencies in medical question texts.

The strength of the DFF-SDQC model lies in its dual-channel architecture, which integrates embeddings of different granularities with the interrogative pronoun attention mechanism. This design effectively leverages the structural and semantic information present in questions, thereby enhancing classification accuracy. This study provides a novel perspective and methodology for question classification and contributes to advancing natural language processing research in the Kazakh language.

#### 3.3.1. CINO pretrained model.

Pretrained language models are trained on large-scale unlabeled datasets to learn general linguistic knowledge or patterns, which can subsequently be fine-tuned for specific downstream tasks. CINO [37] (Chinese mINOrity pretrained language model) is a minority language pretrained model released by the Harbin Institute of Technology and iFLYTEK Joint Laboratory (HFL). CINO is further trained based on XLM-R and employs a method called Cross-Lingual Context Alignment, which leverages both monolingual and bilingual data while considering cross-lingual similarities and differences. CINO has been pretrained on seven languages, including Tibetan, Mongolian, Uyghur, Kazakh, Korean, Zhuang, and Cantonese. For Kazakh, which exhibits complex morphological features, CINO effectively addresses the issue of data sparsity. For a given question containing n words, denoted as $\text{S}=\{{\text{x}}_{\text{1}},x_{\text{2}},\ldots ,x_{n}\}$, each word ${\text{ x}}_{i}$ is input into the CINO model. The input embeddings consist of two components: token embeddings representing the semantic vector of each word and position embeddings representing the word's position in the sequence:


$${\;x}_{i}={token}_{i}+{pos}_{i},$$ (1)

After passing through N Transformer layers, the model produces contextualized word embeddings:


$${\;e}_{i}=CINO({\;x}_{i})$$ (2)

To further adapt these embeddings to downstream cross-lingual question understanding tasks, the CINO model is fine-tuned on the task-specific training dataset. Special tokens, such as [/s], which do not carry intrinsic semantic meaning, are processed by CINO to produce output representations that better capture the overall semantics of the input sequence. The resulting embeddings e are then fed into a BiLSTM module for subsequent bidirectional contextual feature extraction.

#### 3.3.2. BLSTM.

Long Short-Term Memory (LSTM) networks exhibit strong capabilities in sequential modeling, enabling the capture of contextual information across long distances. To effectively model word order and context-dependent features, a bidirectional LSTM (BiLSTM) layer is employed. In this layer, a weight-sharing mechanism is used to share parameters between question classification and named entity recognition tasks, thereby enhancing feature representations.

Specifically, the output embeddings from the CINO model,${\;e}_{i}$ are fed into the BiLSTM to generate a sequence of hidden vectors $h_{\text{1}}$,$\;h_{\text{2}}\ldots h_{i}\ldots h_{n}$, encoding the contextual information of the entire question S into $h_{i}$. The forward and backward hidden states are computed as follows:


$$h_{f}=\overset{\mathrm{\backslash relbar}\;\;\mathrm{\backslash relbar}\;\;\mathrm{\backslash relbar}\;\;\mathrm{\backslash relbar}\;\;\mathrm{\backslash relbar}\;\;\mathrm{\backslash relbar}\;\;⇀}{LSTM}\left({\;e}_{i},\beta \right),\;i\in [\text{1},n]$$ (3)

$$h_{b}=\overset{↼\;\;\mathrm{\backslash relbar}\;\;\mathrm{\backslash relbar}\;\;\mathrm{\backslash relbar}\;\;\mathrm{\backslash relbar}\;\;\mathrm{\backslash relbar}\;\;\mathrm{\backslash relbar}\;\;}{LSTM}\left({\;e}_{i},\beta \right),\;i\in [\text{1},n]$$ (4)

$$h_{i}=\{\overset{f}{\underset{\text{1}}{h}}:\overset{b}{\underset{\text{1}}{h}},\overset{f}{\underset{\text{2}}{h}}:\overset{b}{\underset{\text{2}}{h}},\ldots \overset{f}{\underset{i}{h}}:\overset{b}{\underset{i}{h}}\ldots \overset{f}{\underset{n}{h}}:\overset{b}{\underset{n}{h}}\}$$ (5)

Here, $h_{f}$ and $h_{b}$ represent the forward and backward hidden states, respectively; β denotes the LSTM model parameters, and $h_{i}$ is the output vector of the BiLSTM layer, which captures the bidirectional contextual features for each word in the question sequence.

#### 3.3.3. Construction of the Interrogative Pronoun Attention Matrix.

In question classification, interrogative pronouns exert a significant influence on task performance, aiding in the identification of question type, intent, and grammatical structure. In this study, an interrogative pronoun attention matrix is employed to enhance semantic information in questions, thereby improving the model’s ability to assess the impact of interrogative words. By integrating word embeddings with a diagonal attention matrix focused on interrogative pronouns, the model can identify which portions of a question are most relevant to the interrogative terms.

**Construction of an Interrogative Pronoun Lexicon**. Based on collected questions and Kazakh grammar references, a lexicon of 160 Kazakh interrogative pronouns was compiled. These pronouns are frequently used in sheep disease questions and exhibit distinct characteristics across different question types.

**Identification of Interrogative Pronouns in Questions**. Using the lexicon, interrogative pronouns are located within each question. For example, in the question “قويدىڭ سىرەسپەسى قانشالىق ۋاقىتتا جازىلادى؟” (“What is the treatment duration for sheep tetanus?”), the interrogative pronoun e is “قانشالىق” (“how long”). First, word embeddings $w_{n}$ are trained at the word level, as detailed in the following section. Then, a diagonal attention matrix $\overset{r}{\underset{i}{A}}$ is computed to capture the semantic relevance between each word $w_{i}$ and the interrogative pronoun e, defined as:


$$\overset{r}{\underset{i}{A}}=f(e,w_{i})\times \beta$$ (6)

where f denotes the inner product function and β is a learnable parameter. For each element in the diagonal attention matrix, the relative importance $\overset{r}{\underset{i}{\alpha }}$ of the word $w_{i}$ with respect to eee is calculated as:


$$\overset{r}{\underset{i}{\alpha }}=\frac{exp(\overset{r}{\underset{i}{A}})}{\sum_{j=\text{1}}^{n}exp(\overset{r}{\underset{j}{A}})}$$ (7)

the attention-weighted word embeddings incorporating interrogative pronoun are computed as:


$$x_{i}=\overset{r}{\underset{i}{\alpha }}\times w_{i}$$ (8)

the resulting interrogative pronoun attention matrix is constructed as:


$${X=[x}_{\text{1}},x_{\text{2}},\ldots ,x_{n}]$$ (9)

Integrating the interrogative pronoun attention mechanism enables the intent classification model to more accurately identify the segments of a question that exert the greatest influence on classification, thereby enhancing overall model performance.

#### 3.3.4. CNN network with attention-based pooling.

Convolutional Neural Networks (CNNs) have been widely demonstrated to extract high-dimensional local features from raw data through convolution operations, which play a critical role in recognition and classification tasks. In this study, the output of the interrogative pronoun attention matrix, ${X=[x}_{\text{1}},x_{\text{2}},\ldots ,x_{n}]$, is fed into a CNN for convolutional feature extraction. CNN employs convolutional kernels of sizes k=2,k=3,k=4 to capture local information of varying granularities within the question. The convolution operation produces a new feature$y_{i}$, defined as:


$$y_{i}=f\left(w_{k}\cdot x_{i;i+k-\text{1}}+b\right),\;k=\text{2},\text{3},\text{4}$$ (10)

where f denotes the nonlinear activation function (ReLU is used in this study),$\;w_{k}\in R^{k\times d}$ represents the convolution kernel with size kand word embedding dimension d, “·” denotes the dot product, and $y_{i}$ is the resulting feature for the i convolution operation. For a question of length nnn, the convolutional feature vector is expressed as:


$$Y=\;{[y}_{\text{1}},y_{\text{2}},\ldots ,y_{n-k+\text{1}}]$$ (11)

Typically, pooling operations are applied following convolution to reduce feature dimensionality and extract salient information. Conventional pooling methods, such as max pooling and average pooling, have inherent limitations: max pooling may discard non-maximal activations, resulting in information loss, while average pooling may dilute strong activation signals due to averaging. To address these issues, this study introduces attention-based pooling (Att-pooling) and Top_k pooling. These approaches effectively mitigate the loss of critical feature information. The detailed procedure is as follows:

**Attention-based Pooling.** Compute hidden units $U_{t}$ using the nonlinear activation function tanh:


$$U_{t}=tanh(U_{w}Y+b_{w})$$ (12)

where Y is the convolutional feature vector, $U_{w}$ as a learnable context vector and $b_{w}$ is the bias vector. Then calculate attention probabilities $a_{t}$ using the Softmax function:


$$a_{t}=Softmax(\overset{T}{\underset{t}{U}}+U_{w})$$ (13)

the attention-pooled value $P_{ai}$ is then obtained by the weighted summation of hidden units:


$$P_{ai}=\sum_{t}a_{t}h_{t}$$ (14)

the resulting feature vector is expressed as:


$$P_{a}=\;{[P}_{a\text{1}},P_{a\text{2}},\ldots ,P_{an}]$$ (15)

**Top_k Pooling.** On the basis of Att-pooling, the Top_k pooling selects the klargest values as the final output features. Compared to traditional max pooling or average pooling, Top_k pooling preserves the most critical local convolutional features relevant for question classification. The computation is defined as:


$$P_{ti}=\;top\_k(P_{a})$$ (16)

where, k is set to 2, and the final feature representation obtained via Top_k pooling is denoted as:


$$U=[P_{t\text{1}},P_{t\text{2}},\ldots ,P_{tn}]$$ (17)

The combination of Att-pooling and Top_k pooling offers several advantages: first, Att-pooling reweights convolutional features according to their importance, enabling a more precise understanding of the question's intent. Compared to average pooling, Att-pooling better preserves high-intensity local features. Second, Top_k pooling retains more critical convolutional features than conventional max pooling, which is particularly beneficial for question classification. Finally, the combined pooling approach effectively minimizes the risk of information loss.

#### 3.3.5. Dual-channel feature fusion.

The final classification layer of the question classification model employs a concatenation operation followed by a Softmax function to predict the question type. Specifically, the model integrates the outputs of a dual-channel hybrid neural network consisting of an attention-pooled CNN channel and a BiLSTM channel. The CNN channel produces a fixed-length feature vector U through Top_k pooling. In parallel, the BiLSTM processes the input sequence and generates a sequence of hidden states; to ensure dimensional consistency for feature fusion, a time-wise pooling operation is applied to the BiLSTM hidden state sequence, yielding a fixed-length semantic feature vector S The fused representation is then obtained by concatenating the two vectors:


Y=concat(U,S) (18)

where the concatenation is performed along the feature dimension. Finally, the Softmax function is applied to Y to compute the probability distribution over different question types. The Softmax operation is defined as:


$$\hat{y}=softmax(W_{f}\cdot Y+b_{f})$$ (19)

where $\hat{y}$ denotes the predicted probability vector of question categories in the DFF-SDQC model, $W_{f}$ is the weight matrix of the fully connected layer, and $b_{f}$ is the bias vector. The Softmax function allows for the computation of a normalized score distribution across categories, thereby enhancing classification discriminability. Furthermore, the cross-entropy loss function is employed to optimize the model parameters, defined as:


$$loss=\;-\sum_{i=\text{1}}^{T}\sum_{j=\text{1}}^{C}y_{i}log\overset{j}{\underset{i}{\hat{y}}}+\lambda ∥\theta {∥}^{\text{2}}$$ (20)

where T represents the total number of training samples, C denotes the number of question categories, $y_{i}$ is the ground-truth label of the i sample, λ is the regularization coefficient, and θ denotes the trainable parameters of the model. This loss formulation ensures both accurate classification and model regularization, promoting generalization performance.

## 4. Experiments and analysis

### 4.1. Datasets

#### 4.1.1. D-SDQC.

The D-SDQC dataset was constructed in this study specifically for sheep disease question classification. It comprises seven primary categories: Disease Information, Symptoms, Diagnosis, Treatment, Prevention, Husbandry Management, and Emergency Care. Additionally, it includes 32 subcategories, such as Disease Definition, Etiology, Type, Disease Stage, Disease Symptoms, Symptom Trends, Symptom-Related Factors, Clinical Diagnosis, Required Diagnosis, Diagnostic Basis, Complications, Treatment Methods, Recommended Medication, Dosage, Medication Duration, Treatment Effectiveness, Preventive Measures, Vaccine Types, Vaccination Timing, Vaccination Methods, Vaccine Effectiveness, Vaccine Precautions, Environmental Sanitation, Feed and Water, Quarantine and Inspection, Seasonal Prevention, Preventive Guidelines, Husbandry Experience, Emergency Measures, Emergency Medication, Quarantine Measures, and Transmission Routes. To ensure a fair and reliable evaluation, the dataset was processed following a strict split-before-augmentation strategy. Specifically, the original question corpus was first divided into training, validation, and test sets at a ratio of 8:1:1. Subsequently, data augmentation techniques based on synonym substitution and paraphrase generation were applied only to the training set to enhance data diversity and improve model generalization ability. The validation and test sets consisted exclusively of original, non-augmented questions. After augmentation, the final dataset contained a total of 7,000 question instances, which were used for model training and evaluation.

#### 4.1.2. D-TQC.

Given that Kazakh is a low-resource language, publicly available datasets are generally limited. To enhance experimental diversity and reliability, the tourism question classification dataset D-TQC [22] was employed to further evaluate the performance of the DFF-SDQC model and to conduct comparative experiments.

The D-TQC dataset consists of six primary categories: Entity, Time, Description, Numeric, Location, and Person. It includes 26 subcategories, such as Route, Specialty, Cuisine, Cultural Customs, Transportation, Climate Type, Accommodation, Time Period, Time Point, Reason, Abbreviation, Alias, Review, Distance, Price, Contact Information, Altitude, Area, Scenic Spot Level, Temperature, Administrative Region, Scenic Spot, Address, Specific Tasks, Personal Description, and Group/Organization. The dataset contains a total of 9,211 question instances. For the experiments, the D-TQC dataset was also split into training, validation, and test sets at an 8:1:1 ratio.

### 4.2. Baselines

To evaluate the performance of the proposed question classification model on both the self-constructed sheep disease question dataset (D-SDQC) and the tourism question classification dataset (D-TQC), several baseline models were selected for comparison.

● SVM [20]: As a traditional machine learning algorithm, Support Vector Machines (SVM) have been widely applied in various text classification tasks. Wang Hua et al. were among the first to apply the SVM model for Kazakh text classification, achieving promising results.
● CNN [21]: Shardal et al. employed Convolutional Neural Networks (CNN) to address Kazakh short-text classification. This approach first represents words or stems as vector embeddings using Word2Vec, followed by classification using a CNN architecture.
● BiLSTM+CNN [12]: This model first extracts local features of the question using a CNN, followed by a BiLSTM network to capture global contextual information across the entire question.
● BiGRU + CNN [4]: Utilizing a bidirectional Gated Recurrent Unit (BiGRU) network, this model captures semantic relationships within the question while a CNN is applied to enhance the understanding of local semantic features.
● BiGRU + CNN + Att+Features [23]: By integrating various lexical and syntactic features, such as stems, affixes, part-of-speech tags, and phrase-level representations, this model effectively combines them with a BiGRU + CNN+Attention deep learning network.
● BiGRU + CNN+INatt [22]: This model represents textual features using both word stem information and an interrogative-word attention mechanism, followed by BiGRU and CNN networks to extract deeper semantic features.
● Transformer [37]: Based on the Multi-Head Attention mechanism, the Transformer model employs its encoder component to encode the semantic information of questions for classification purposes.

### 4.3. Implementation details

The DFF-SDQC model was implemented on the following hardware configuration: GPU acceleration library CUDA 10.1, CPU Intel Core i7, and graphics processing unit NVIDIA GeForce GTX 1660 Ti. The main model hyperparameters are summarized in Table 3.

**Table 3:** Hyperparameter setteings.

Parameter	Value	Parameter	Value
Hidden layer dimension	1024	Dropout	0.5
BiLSTM dimension	256	Optimizer	Adam
Convolution kernel sizes	{2,3,4}	Text length	128
Learning rate	0.0001	Epochs	12

### 4.4. Evaluation measures

The performance of the question classification models was evaluated using Precision, Recall, and F1-score metrics. Precision measures the proportion of correctly predicted instances among all instances predicted as positive for a given class, defined as:


$$Precision=\frac{TP}{TP+FP}$$ (21)

Recall measures the proportion of correctly predicted instances among all actual positive instances, defined as:


$$Recall=\frac{TP}{TP+FN}$$ (22)

F1-score provides a harmonic mean between Precision and Recall, balancing the trade-off between these two metrics, and is defined as:


$$F\text{1}=\frac{\text{2}*(precision*recall)}{precision+recall}$$ (23)

### 4.5. Results and analysis

#### 4.5.1. Experiment results on D-SDQC.

The performance of the proposed DFF-SDQC model was evaluated on the D-SDQC dataset and compared with several baseline models. The experimental results are presented in Table 4.

**Table 4:** Experimental Results of Various Models on the D-SDQC Dataset.

Model	P(%)	R(%)	F1(%)
SVM [20]	83.74	83.18	83.46
CNN [21]	86.02	85.46	85.74
BiGRU + CNN [4]	87.82	87.64	87.73
BiLSTM+CNN [12]	87.18	88.80	87.98
BiGRU + CNN + Att+Features [23]	89.09	89.15	89.12
BiGRU + CNN+INatt [22]	90.76	91.16	90.96
Transformer [37]	87.42	88.60	88.01
DFF-SDQC	93.10	93.26	93.18

From the results, it can be observed that traditional machine learning methods, such as Support Vector Machine (SVM), perform significantly worse than deep learning models on the D-SDQC question classification dataset. This performance gap is primarily due to the independence assumption of words in statistical methods, which may fail to effectively capture contextual information in questions.

The CNN-based stem embedding representation slightly outperforms the word embedding method, indicating that effective morphological analysis and stem extraction can enhance text classification performance, particularly in agglutinative languages like Kazakh. BiGRU outperforms CNN, likely because BiGRU can better capture long-term dependencies and contextual information in the text. Moreover, the combined CNN-BiGRU model surpasses either single BiGRU or CNN models, demonstrating the advantage of integrating local feature extraction with sequential temporal features.

The Transformer baseline is a vanilla Transformer model trained from scratch on the D-SDQC dataset. While it leverages positional encoding and multi-head attention to capture contextual relations, its performance is limited by the relatively small dataset size and absence of pre-training.

In contrast, the proposed DFF-SDQC model employs CINO, a pre-trained transformer, as one channel of its dual-channel architecture. Combined with the CNN-based channel and the interrogative pronoun attention mechanism, DFF-SDQC effectively learns both word-level and sentence-level contextual semantic features. This results in a substantial performance improvement over all baselines. These results highlight the complementary benefits of pre-training and dual-channel design for domain-specific question classification in the sheep disease context.

**Computational Cost and Efficiency.** The proposed DFF-SDQC model employs a dual-channel feature fusion architecture, integrating a CINO encoder, BiLSTM, interrogative-pronoun attention, and a multi-scale CNN. While the CINO encoder captures rich Kazakh semantic representations, its parameters are fine-tuned, significantly reducing training overhead compared Transformer models. The BiLSTM module captures bidirectional contextual dependencies with complexity $O(L\cdot d^{\text{2}})$, while the CNN extracts multi-scale features efficiently, and the attention mechanism selectively emphasizes interrogative pronouns. Given the relatively short length of Kazakh questions, the overall computational cost remains manageable.

In terms of parameter size and inference efficiency, The dual channels allow parallel feature extraction, improving GPU utilization and inference speed. Compared to traditional models like SVM, DFF-SDQC incurs higher computation at inference but offers superior semantic modeling, while maintaining significantly lower cost and latency than Transformer-based classifiers. DFF-SDQC achieves a favorable balance between computational efficiency and classification performance, making it suitable for domain-specific Kazakh question classification tasks.

#### 4.5.2. Experiment results on D-TQC.

The experimental results on the public dataset are presented in Table 5, where the DFF-SDQC model demonstrates excellent performance on the D-TQC dataset. Moreover, it can be observed that the proposed dual-channel feature fusion network, significantly contribute to improving the performance on the D-TQC Dataset. These findings validate the effectiveness of the DFF-SDQC model, indicating that it can achieve satisfactory results even under conditions of relatively limited data resources.

**Table 5:** Experimental results of various models on the D-TQC dataset.

Model	P(%)	R(%)	F1(%)
SVM [20]	85.73	85.40	85.56
CNN [21]	91.01	90.74	90.87
BiGRU + CNN [4]	92.53	92.45	92.49
BiLSTM+CNN [12]	91.38	91.77	91.57
BiGRU + CNN + Att+Features [23]	92.78	93.40	93.09
BiGRU + CNN+INatt [22]	94.41	94.35	94.38
Transformer [37]	92.80	92.88	92.84
DFF-SDQC	95.86	96.19	96.02

The experimental results on the public D-TQC Dataset, where the DFF-SDQC model demonstrates excellent performance on the D-TQC dataset. Moreover, it can be observed that the proposed dual-channel feature fusion network, significantly contribute to improving the performance on the D-TQC Dataset. These findings validate the effectiveness of the DFF-SDQC model, indicating that it can achieve satisfactory results even under conditions of relatively limited data resources.

#### 4.5.3. Ablation Study.

To verify the contribution and collaborative effectiveness of each core module, ablation experiments were conducted on the D-SDQC and D-TQC datasets. The results are presented in Table 6 and Table 7 (where √ indicates that the module is enabled and × indicates that it is disabled). Among them, INatt denotes the interrogative word feature fusion module, CINO represents the CINO-based text feature representation module, and BiLSTM refers to the BiLSTM encoding module.

**Table 6:** Ablation experimental results on the D-SDQC dataset.

INatt	CINO	BiLSTM	P(%)	R(%)	F1(%)
√	√	×	87.15	86.59	86.87
×	√	√	92.22	92.65	92.43
√	×	√	91.3	91.82	91.56
√	√	√	93.1	93.26	93.18

**Table 7:** Ablation experimental results on the D-TQC dataset.

INatt	CINO	BiLSTM	P(%)	R(%)	F1(%)
√	√	×	91.62	90.4	91.01
×	√	√	94.77	94.69	94.73
√	×	√	93.16	93.55	93.35
√	√	√	95.86	96.19	96.02

When all three modules—**INatt**, **CINO**, and **BiLSTM**—were enabled, the DFF-SDQC model achieved the best evaluation metrics and overall performance. This indicates that the three modules are non-conflicting and can effectively cooperate to enhance the model’s capability. When any one of the modules (INatt, CINO, or BiLSTM) was disabled, the performance of the DFF-SDQC model decreased to varying degrees. Specifically, disabling the INatt module led to a relatively small performance drop, while removing the BiLSTM module caused a more significant decline. These results demonstrate that the INatt, CINO, and BiLSTM modules complement each other well and collaboratively improve the model’s performance by enabling effective learning across different feature modalities, thereby substantially enhancing the overall model performance.

#### 4.5.4. Experimental results on varying sentence lengths.

The majority of questions in the sheep disease domain dataset are relatively short. To evaluate whether incorporating a question-word attention mechanism can enhance classification performance for short texts, comparative experiments were conducted using question sets of different lengths. Specifically, the dataset was divided into two subsets based on the number of words per question: questions containing nine words or fewer were considered short questions, while questions containing ten words or more were classified as long questions.

From the results in Table 8, it can be observed that, this division allows for a systematic assessment of the model’s effectiveness across varying text lengths and provides insights into the impact of attention mechanisms on short versus long questions.

**Table 8:** This is a table. Tables should be placed in the main text near to the first time they are cited.

Sentence type	Interrogative Words(w/o)	P(%)	R(%)	F1(%)
Short sentence	×	91.09	90.65	90.87
	√	92.09	91.93	92.01
Long sentence	×	91.29	91.31	91.30
	√	92.01	92.37	92.19

## 5. Conclusion

In this study, we proposed a Dual-Channel Feature Fusion Network for Sheep Diseases Question Classification (DFF-SDQC). The model leverages the CINO pre-trained language model and Interrogative Pronoun Attention to extract rich semantic representations of sheep disease-related questions. Subsequently, CNN and BiLSTM networks are employed to capture both local and global textual features of the questions. The extracted features are then integrated using a proposed feature fusion mechanism and fed into a fully connected layer for classification. Experimental evaluations were conducted on two datasets: a self-constructed sheep disease question classification dataset (D-SDQC) and a tourism question classification dataset (D-TQC). The results demonstrate that DFF-SDQC achieves high classification accuracy, outperforming several baseline models. Despite its effectiveness, the proposed model has certain limitations. Future work will focus on further refining the feature fusion mechanism to make it applicable to other models. Additionally, integrating domain-specific veterinary knowledge graphs or leveraging multimodal fusion techniques will be explored to further enhance the performance of DFF-SDQC on sheep disease question classification tasks. In terms of veterinary and livestock management applications, the proposed DFF-SDQC model can serve as a core component of intelligent question-answering systems by accurately classifying sheep disease related questions. This capability assists veterinarians and farm managers in handling inquiries from farmers. Moreover, the model is capable of understanding domain-specific terminology and contextual information in low-resource languages, which is particularly valuable in rural areas with limited access to professional veterinary resources.
